# A Systematic Review of the Effects of Exercise and Physical Activity on Non-Specific Chronic Low Back Pain

**DOI:** 10.3390/healthcare4020022

**Published:** 2016-04-25

**Authors:** Rebecca Gordon, Saul Bloxham

**Affiliations:** Department of Sport and Health Sciences, University of St Mark and St John, Plymouth PL6 8BH, UK; sbloxham@marjon.ac.uk

**Keywords:** aerobic fitness, non-specific chronic low back pain, pedometer, physical activity

## Abstract

Back pain is a major health issue in Western countries and 60%–80% of adults are likely to experience low back pain. This paper explores the impact of back pain on society and the role of physical activity for treatment of non-specific low back pain. A review of the literature was carried out using the databases SPORTDiscuss, Medline and Google Scholar. A general exercise programme that combines muscular strength, flexibility and aerobic fitness is beneficial for rehabilitation of non-specific chronic low back pain. Increasing core muscular strength can assist in supporting the lumbar spine. Improving the flexibility of the muscle-tendons and ligaments in the back increases the range of motion and assists with the patient’s functional movement. Aerobic exercise increases the blood flow and nutrients to the soft tissues in the back, improving the healing process and reducing stiffness that can result in back pain.

## 1. An Introduction to the Impact of Back Pain on Society and the Importance of Physical Activity

Back pain is a major health issue in Western countries and is associated with increasing medical expenditure, work absence [1,2] and is the most common musculoskeletal condition [3,4,5]. Sixty to eighty percent of adults will at some point in their lives experience low back pain [6,7,8], and 16% of adults in the United Kingdom (UK) consult their general practitioner every year [9]. Back pain costs the National Health Service (NHS) £1.3 million every day [10] and results in 12.5% of all work absence in the UK [11]. However, the most appropriate intervention to treat non-specific chronic low back pain (NSCLBP) remains elusive [12].

It is recommended for patients with NSCLBP to remain physically active, as long periods of inactivity will adversely affect recovery [13,14]. A variety of different types of exercise have been explored to treat CLBP, including low-to-moderate intensity aerobic exercise [15,16], high intensity aerobic exercise [17,18], core stabilization and muscular strength exercises [19,20,21,22,23,24] and flexibility programmes [25,26,27]. However, the most effective form of exercise as a method of rehabilitation for NSCLBP is unknown [6,28] reflecting its complexity [17] and more research is required [29].

Physical activity (PA) to increase aerobic capacity and muscular strength, especially of the lumbar extensor muscles, is important for patients with CLBP in assisting them to complete activities of daily living [30]. However, different exercises have been found to result in varying levels of effectiveness in reducing lower back pain [31]. In addition, too much or too little PA can be associated with low back pain [32], suggesting that PA as an intervention for low back pain is complex.

Eight-five percent of back pain cases have an unknown cause [33], normally diagnosed after undergoing tests such as X-ray, MRI scan and blood tests [34]. Understanding the cause of back pain is important in order to remove it from the patient’s life and not to replicate the movement during therapy [35]. However, when the cause of the back pain is unknown, prescribing targeted therapy can prove difficult, and general exercise is often recommended [36]. Typically intervention programmes have adopted a monodisciplinary approach to rehabilitate NSCLBP [15,21,25]. Although promising findings were reported following a multicomponent exercise programme [37]. Thirty-seven patients with NSCLBP were allocated into control, (who just maintained their current rehabilitation programme), or training groups, which combined an additional functional training programme of aerobic exercise, muscular strength and flexibility. Back pain was found to significantly decrease by 52.5% in the training group compared to no significant change in the control group. In addition, disability significantly decreased by 27.3% in the training group according to the Oswestry Disability Index, compared to no significant change in the control group. The aim of this article is to review the effects of PA and exercise interventions involving aerobic exercise, muscular strength and stabilisation exercises and/or flexibility training on NSCLBP to identify effective strategies for treatment.

## 2. Method

A systematic review was carried out between 2014 and 2015 using the databases SPORTDiscuss, Medline and Google Scholar. The first author selected intervention programmes published between 2005 and 2015 which investigated the effect of PA or exercise interventions for NSCLBP patients involving aerobic exercise, muscular strength and stabilisation exercises and/or flexibility training on NSCLBP. The first author read and reviewed the articles. Chronic pain was defined as pain remaining for longer than three months and further inclusion criteria was that the participants involved in the studies should be ≥18 years old. The intervention programmes were identified using the search terms “non-specific chronic low back pain and exercise” which returned 141 results. Other search terms included “chronic low back pain and aerobic exercise” (187 results), “chronic low back pain and muscular strength” (120 results) and “non-specific chronic low back pain” (173 results). A total of 14 studies were included within the final review. The review summarised the effect on NSCLBP within the included intervention programmes.

### 2.1. Eligibility Criteria

Studies were included within the final review based on the following: population, intervention and the outcome.

### 2.2. Inclusion Criteria

**Population:** NSCLBP patients aged 18 years or older.

**Intervention:** Aerobic exercise, muscular strength or stabilisation exercises and/or flexibility training intervention programmes. There was no restriction on the inclusion of a follow up in the included studies.

**Outcome:** Investigate effect of the intervention on NSCLBP which was not limited to one specific measure for pain.

### 2.3. Exclusion Criteria

Literature reviews and any article which did not involve a delivery of an intervention programme to NSCLBP patients.

## 3. Defining Back Pain and the Impact of Physical Activity and Exercise

Back pain is defined as chronic when the pain remains for longer than three months [38]. CLBP can have a debilitating effect on patients’ lives, resulting in disability and reducing their ability to carry out activities of daily living [29]. Acute back pain is pain that remains for less than 6 weeks [39,40] and sub-acute back pain is back pain for between 6 weeks and 3 months. Forty percent of patients with acute low back pain are at an elevated risk of developing CLBP [41].

Back pain is then further categorised into specific or non-specific back pain. Non-specific back pain is diagnosed when the cause of the back pain is unknown [42,43], and specific back pain refers to a specific cause for the pain, for example an infection or a fracture [44]. Non-specific low back pain is the most common type of back pain to occur [45,46], and accounts for 85% of all back pain cases [39,47].

PA increases the blood flow to the back which is important for the healing process of the soft tissues in the back [48]. Being physically active, through activities of daily living, has been highlighted as important in assisting the recovery of acute and NSCLBP [49]. However, following a review of 39 trials into the effects of exercise on non-specific acute low back pain [2], it was suggested there is strong evidence that an exercise programme was not more effective for recovery of non-specific acute low back pain, compared to inactivity. Thus, patients with acute low back pain should not start an exercise programme for rehabilitation [50].

The difference between PA and exercise is that exercise is planned and structured which involves disrupting homeostasis by concentric, eccentric and isometric muscular activity and involves repetitive movements [51]. PA is not structured, and includes any movement that involves contraction of skeletal muscles requiring energy expenditure [52] typified by activities of daily living such as walking and housework [53].

Most people with non-specific acute low back pain recover in 4–6 weeks with or without a treatment [5]. Therefore if acute low back pain patients recover without a treatment in a similar timescale to patients with a treatment, there is no added benefit in completing an exercise programme such as muscle strengthening exercises. Muscle strengthening exercises could potentially cause extra damage to acute back pain due to the additional strain on the ligaments and muscles in the back, which may have swelling [48]. It is important to stop exercise in order to reduce the swelling of the affected area and therefore reduce the back pain [39], suggesting it is a case of waiting for acute low back pain to recover.

Furthermore, a review of six randomised controlled trials researched the effect of exercise programmes on patients with non-specific sub-acute low back pain [54]. The review suggested that there was moderate evidence that a graded-activity exercise programme is effective for improving absenteeism from work for patients with non-specific sub-acute low back pain, however it was unclear if other types of exercise programmes are effective.

## 4. Results

### 4.1. Aerobic Exercise

Aerobic exercise can benefit CLBP as it increases the blood flow and nutrients to the soft tissues in the back, improving the healing process and reducing stiffness that results in back pain [55]. In addition 30–40 min of aerobic exercise increases the body’s production of endorphins [55], a brain chemical that bind to the opiate receptors in the pain control system in the brain and spinal cord to decrease the perception of pain [56]. Endorphins act in a similar way to pain reducing drugs such as morphine and codeine [57]. However increasing the body’s endorphin production is a natural alternative for pain relief for the body [58], and can reduce CLBP [59]. Rehabilitation involving aerobic exercise can be used as a conservative method for reducing CLBP, and could prevent patients relying on medication for pain reduction.

A low aerobic fitness level is associated with CLBP [60,61], and maximum oxygen consumption (VO_2max_) was significantly lower by 10 mL/kg in men with CLBP compared to men without [62]. VO_2max_ was also significantly lower by 5.6 mL/kg in women with CLBP compared to healthy counterparts.

Aerobic exercise for 20 min on a cycle ergometer at 70% peak oxygen uptake reduced the pain perception for more than 30 min for patients with CLBP [63]. Aerobic exercise also provides additional benefits such as improving functional status [64], and reducing the fear of movement [65]. Fear of movement is a predictor for functional limitations [66] and is associated with disability in patients with CLBP [67]. Aerobic exercise can reduce disability and improve the functional status of patients with CLBP by increasing fitness levels, helping patients conduct activities of daily living.

#### 4.1.1. Impact of Aerobic Exercise Interventions on Chronic Low Back Pain

A 6-week moderate intensity aerobic exercise programme (walking on a treadmill at 50% heart rate reserve) for 52 sedentary NSCLBP patients was compared to a 6-week programme involving specific strengthening exercises for the trunk and upper and lower limbs [16]. CLBP significantly reduced by 20% in the aerobic exercise group and 15% in the muscle strengthening group, although there was no significant difference between the two groups. This suggests that patients could be provided with a choice of which type of exercise programme they would most enjoy. This is important as enjoyment of exercise is an important factor in exercise adherence [68]. However this study involved a 6-week intervention, and an 8-week intervention programme is important to significantly improve aerobic fitness [69], by allowing greater physiological adaptions to occur [15].

An 8-week moderate intensity aerobic exercise intervention at 40%–60% of heart rate reserve combined with conventional physiotherapy, significantly reduced NSCLBP by 47% [15]. This was compared to a significant reduction of 42% in NSCLBP in the control group, involving only conventional physiotherapy. However there was no significant difference between the two groups, suggesting the combination of moderate intensity aerobic exercise and conventional physiotherapy does not provide any additional benefits to CLBP.

The 8-week intervention programme was also found to increase aerobic fitness by 3.3% as measured by VO_2max_. This increase was not significant, and also suggests that additional factors excluding aerobic fitness levels must have had an influence on reducing CLBP. This was in contrast to previous research which suggested aerobic fitness levels to be associated with CLBP [60,61]. The conventional physiotherapy involved activities such as back mobilisation exercises, core stabilisation exercise and education on back care, suggesting a general programme involving a range of activities may be optimal.

A 12-week high intensity aerobic exercise programme involving running on a treadmill at 85% of heart rate reserve and was compared to passive treatment (ultrasound and did not include any form of PA) [17]. The 12-week high intensity exercise programme significantly reduced NSCLBP by 41% compared to no improvement in the passive treatment group.

The effect of high intensity aerobic exercise on CLBP was further supported by a 12-week high intensity aerobic exercise programme (running on a treadmill at 85% heart rate reserve) which significantly reduced NSCLBP by 30% [18]. This study involved a larger sample size of 64 patients, compared to the previous study [17].

However the study [18] excluded patients with NSCLBP who were obese, classified by a body mass index of 30 or over [70,71]. The researcher stated this was due to possible cardiovascular problems and the risk of injury to the patients, as the study involved high intensity exercise. Therefore the results from this study cannot be generalised to obese NSCLBP patients, despite obesity being associated with NSCLBP [72].

Walking is known to be a safe form of exercise for CLBP patients as it is associated with a low injury rate [73] and does not involve twisting or vigorous forward flexion [74]. Although, exercising at a low intensity at 40% VO_2max_ does not significantly increase cortisol levels [75], and low cortisol levels are associated with CLBP [76].

These studies indicate that although similar outcomes can be achieved despite differences in aerobic exercise intensity. Thus moderate exercise should be promoted over high or low intensity programmes given the reduced risks, enhanced compliance, optimal benefits and reduced impact [55].

Exercising at a comfortable intensity for the patient is important in reducing fear avoidance [77], which is important for increasing PA levels [78] as CLBP patients who are more fear avoidant report higher levels of disability [79]. Patients should be encouraged to increase their levels of PA at an intensity that is comfortable for them, and that can be integrated into activities of daily living [53]. Such an approach is more sustainable long term [80]. See Table 1 for a summary of each of the discussed aerobic exercise intervention programmes.

#### 4.1.2. Summary

Moderate intensity aerobic exercise (40%–60% heart rate reserve) should be promoted for NSCLBP rehabilitation. Aerobic fitness, behavioural treatment and multi-disciplinary treatment programmes are important for reducing CLBP and improving disability [81].

### 4.2. Muscle Strength and Stabilisation Training

A reduction in core strength can lead to lumbar instability [82], and lumbar instability also reduces the flexibility of the lumbar spine [83]. CLBP patients restrict their trunk movement to reduce the pain in the lumbosacral area, however this only further reduces core strength and increases lumbar instability, resulting in low back pain [84]. Exercises to activate the deep abdominal muscles including the superficial muscles, transversus abdominis muscle and the multifidus are important for CLBP patients [85]. The deep abdominal muscles are essential for supporting the lumbar spine and strengthening these muscles can reduce back pain [86].

A high volume of stress placed on the vertebral column muscles can lead to back pain [87], and poor muscle recruitment of the deep abdominal muscles has been shown in NSCLBP patients [19]. The transversus abdominis is important in muscular stabilisation of the spine which assists in supporting posture [88] and a delayed muscle contraction during movement is often prevalent in patients [89]. Spinal stabilisation exercises aim to increase the strength and endurance of these muscles [90], improving spine stability [91].

Stabilisation exercises have been shown to be effective in reducing NSCLBP [19,21,24], but not acute low back pain [92]. It is important to identify the specific exercises which are most effective for a specific population, as opposed to a generic group [93]. Lumbar stabilisation programmes increase the stability of the spine by training the muscular motor patterns in order to reduce low back pain [94].

Strengthening exercises are considered the most effective treatment for functional gain including walking speed [16]. This is because the deep trunk muscles are active when walking [16], suggesting that strengthening these muscles can help with completing activities of daily living [95].

#### 4.2.1. Muscular Strength and Stabilisation Intervention Programmes

Core stabilisation programmes [19,21,23,24] have been shown to significantly reduce CLBP by 39%–76.8%, and a muscular strength programme significantly reduced CLBP by 61.6% [20].

A 3-month intervention involving 30 NSCLBP patients compared core stabilisation exercises including slow curl ups, bird dog, the plank and sit ups (raising the head and shoulders off the ground with the hands under the head) to conventional spine exercises [19]. The conventional spine exercises included static stretching of muscles found to be tight, however the study does not state which form of assessment was used to identify tight muscles.

Core stabilisation exercises significantly reduced NSCLBP by 76.8% compared to a 62.8% significant reduction following the conventional exercises. These findings suggested both core stabilisation and conventional exercises to be significantly beneficial in reducing CLBP. However the core stabilisation group reported a significantly greater improvement compared to conventional exercises, highlighting the importance of core stability for CLBP patients.

An 8-week core stability intervention programme for 10 NSCLBP patients involved activating core stability responses using unstable standing surfaces and unexpected movements of the upper limbs [21]. CLBP significantly reduced by 39.5%. These results were lower in comparison to the other study [19] which reported a 76.8% significant decrease in CLBP. However this study involved a 3-month intervention [19] compared to the 8-week core stability intervention [21], suggesting the longer a stabilisation intervention programme is, the more positive impact upon CLBP there is.

Another study involved an 8-week stabilisation programme [24] involving 40 NSCLBP patients and investigated the effects of combining ankle dorsiflexion exercises with drawing in the abdominal wall (experimental group), to drawing in the abdominal wall exercises alone (control group). The ankle dorsiflexion exercises were completed at 30% of maximal voluntary isometric contraction of the tibialis anterior muscle, using a resistance band for 10 sets of 20s.

Ankle dorsiflexion exercises were included in the exercise programme because the proprioceptive neuromuscular facilitation irradiation technique, increases core muscular strength by stimulating stronger muscles from the lower body [96], which provides a resistance and stimulus to increase muscle fibres and muscle activity in abdominal muscles [97]. This suggests that to contract the deep target muscle the transversus abdominis, resistance should be applied to the stronger ankle dorsiflexors combined with drawing in the abdominal wall. The transversus abdominis and internal oblique muscles are important for core stability as they are attached to the thoracolumbar fascia, and increase the stiffness of the tissue which improves the core stability [98]. In addition an increase in the stiffness of the tissue in the core, can help to resist the stress placed on the spine and help to reduce back pain [99].

The study reported that the experimental group significantly reduced NSCLBP by 32.5% (according to the VAS), 23.2% (Pain Disability Index) and 21.5% (Pain Rating Scale). The control group significantly reduced CLBP by 16.8% (VAS), 12.4% (Pain Disability Index) and 8% (Pain Rating Scale).

This study [24] also included a follow up measurement after 2 months, in which time patients were instructed to continue the exercises of combining ankle dorsiflexion to drawing in the abdominal wall (experimental group), or only drawing in the abdominal wall (control group). The results identified CLBP had significantly reduced further to 46.8% (VAS), 39.2% (Pain Disability Index) and 30.7% (Pain Rating Scale) in the experimental group and 38.7% (VAS), 18.8% (Pain Disability Index) and 14.6% (Pain Rating Scale) in the control group. These results provide additional support for the benefits of a longer intervention programme and also for the inclusion of ankle dorsiflexion exercises in rehabilitation of NSCLBP.

Core stability measured by the active straight leg raise was also shown to improve by 56.1% in the experimental group, and 27.4% in the control group after 8 weeks. The results highlighted the importance of core stability in reducing CLBP, especially as core stability had improved by an additional 33.8% at the two month follow up compared to the 8-week measurement in the experimental group, and consequently CLBP had been shown to reduce further. Therefore the results suggested that the addition of ankle dorsiflexion exercises when combined with drawing in the abdominal wall to be an effective exercise in reducing CLBP.

The addition of ankle dorsiflexion exercises to drawing in the abdominal wall is a unique technique for improving core stability for NSCLBP patients, as this technique has only been previously researched in 40 healthy participants [100]. This study [100] reported that the combination of drawing in the abdominal wall and ankle dorsiflexion exercises, resulted in a significantly greater increase in the thickness of the transverse abdominal muscle measured using ultrasonography, compared to drawing in the abdominal wall alone. This is important for improving core strength [101].

The importance of core stability and muscular strength was emphasised by research which had reported that a slumped sitting posture involving lumbar flexion, resulted in a lower activation of the core muscles such as the lumbar multifidus, iliocostalis lumborum pars thoracis and the transverse fibers of internal oblique [102]. Consequently the muscles become weaker which negatively impacts upon the ability to maintain an upright posture [103]. This is because the intervertebral discs are composed of the annulus fibrosus, which connects the spinal vertebrae above and below the disk [104]. The annulus fibrosus requires a highly structured organisation involving aligned collagen fibres within the transverse axis of the spine, which forms an angle-ply laminate structure [105]. However, when the intervertebral disk degenerates the annulus fibrosus becomes unorganised, which can result in low back pain [106]. This is due to mechanical and structural problems such as tears and delamination [107], as the annulus fibrosus distributes force on the intervertebral discs to prevent the gelatinous material in the soft inner core of the intervertebral disc from leaking out [104].

Patients with low back pain adopt a sitting posture with significantly more lumbar flexion than those without low back pain [108,109]. Therefore this suggested a relationship between a poor sitting posture and low back pain, and highlighted the importance of improving core strength and stability [86] to support an upright sitting posture. In contrast no relationship was reported between low back pain and lumbar flexion when sitting in 170 female undergraduate nursing students, with either minor or significant low back pain or without lower back pain [110]. However this study involved only female participants, and males had been shown to be more associated with lumbar flexion when sitting, with an average of 12.2° more flexion than females [103].

A 12-month exercise programme focused on increasing control of the lumbar neutral zone [23] and involved 106 middle aged working men who had a reported episode of non-specific low back pain within the previous 3 months, but did not have severe disability. The participants exercised twice a week undergoing exercises which aimed to improve lumbar stability, such as abdominal curl up with slight rotation and squat exercises. This exercise programme was combined with educating the patients on back pain and providing training on correct techniques for lifting. Low back pain significantly decreased by 39%, suggesting exercises focusing on lumbar stability combined with education to be effective at reducing low back pain. However it was suggested that the participants may have reported a reduced lower back pain as they knew they were involved in the intervention group and therefore expected to experience less back pain [23].

A muscular strength 8-week intervention programme involving 47 women with NSCLBP [20] investigated the effect of different angles of inversion traction on muscular strength and NSCLBP. The study reported that the inversion −30° group and inversion −60° group was more effective at reducing NSCLBP, and improving core muscular strength than the supine group. NSCLBP significantly reduced by 61.6% in both the inversion −30° group and inversion −60° group, compared to 34.9% in the supine group. In addition extensor back muscle strength was also found to increase by 22.5% (inversion −30° group) and 47% (inversion −60° group), however muscular strength was found to reduce by 6% in the supine group. This suggested that another factor other than muscular strength influenced the decrease in back pain for the supine group.

Trunk extension flexibility was also shown to improve in all three groups. However the biggest increase of 22% was reported in the inversion −60° group compared to an increase of 13.3% in the inversion −30° group, and 4.8% in the supine group. This suggested that a range of factors are responsible for the decrease in NSCLBP, and indicates that a general intervention programme focusing on a range of different areas of fitness is important for NSCLBP rehabilitation.

A 4-week core muscular strength programme (control group) was compared to a core stability programme in addition to core muscular strength exercises (experimental group), in 160 patients with NSCLBP [22]. NSCLBP significantly reduced in the experimental group by 35% compared to 14% in the control group. The results suggested that an intervention programme for NSCLBP which incorporates both core stability and core muscular strength exercises, is more effective at reducing NSCLBP than muscular strength exercises alone.

Four variables exist which may determine the success of a stabilisation exercise programme for CLBP [94]. The four variables include age as participants under the age of 40 have been shown to have higher odds by 3.7 of the stabilisation treatment being a success, an active straight leg raise test higher than 91°, the presence of aberrant movement during lumbar range of motion and a positive prone instability test. Three or more of the four named variables being present is a predictor for the stabilisation exercise programme being successful in reducing CLBP. Therefore it is important to consider the four variables when designing an intervention programme involving stabilisation exercises for CLBP.

Finally, a 15-item questionnaire on clinical instability has been identified [111], which revealed whether patients with NSCLBP respond better to motor control exercises to increase the activation of muscles, including the transversus abdominis, multifidus, and pelvic-floor muscles, or graded activity involving submaximal exercises to increase exercise tolerance. This suggests the questionnaire can help to identify the most effective form of rehabilitation for NSCLBP patients. See Table 2 for a summary of each of the discussed muscular strength and stabilisation intervention programmes.

#### 4.2.2. Summary

Increasing the strength of deep abdominal muscles and improving the stabilisation of the spine is effective at reducing NSCLBP. A core stabilisation programme combined with muscular strength should be considered for NSCLBP patients, as this was shown to be more effective than core muscular strength exercises alone [22]. This suggested a more general programme as opposed to focusing on one particular area of fitness to be more effective at reducing NSCLBP.

### 4.3. Flexibility Training

Stretching the soft tissues in the back, legs and buttock such as the hamstrings, erector muscles of the spine and hip flexor muscles, ligaments and tendons can help to mobilise the spine, and an increase in the range of motion of the spine can assist back pain [112]. This is because stretching can improve the flexibility of the muscle-tendons and ligaments in the back, which is important to increase the range of motion of the joints [113].Therefore an improved range of motion assists with patients’ movement and ability to complete activities of daily living, as most everyday tasks such as lifting and bending require trunk flexion, which involves a complex movement combining lumbar and hip motion [114]. Also stretching exercises decrease the muscle stiffness as a result of changes in viscoelastic properties, due to the decreased actin-myosin cross-bridges and the reflex muscle inhibition [113].

According to the pelvic cross syndrome theory, muscle abnormalities in the postural muscles such as a decreased flexibility and shortening of the hip flexor and back extensor muscles, can result in additional mechanical stress to the joints and soft tissue of the lumbar spine, and can cause lumbar lordosis [87]. Lumbar lordosis is an excessive inward curving of the lumbar spine [115], as a weakening of the abdominal muscles can tilt the pelvis posteriorly, and can result in CLBP [116,117]. In addition the pelvic cross syndrome theory states that hamstring muscle shortening is also important in controlling lumbar lordosis [87]. Hamstring muscle shortening reduces the hip flexion range of motion due to being attached to the posterior leg and the ischial tuberosity, which can affect the lumbopelvic movement during forward bending and can cause low back pain [114].

Flexibility exercises are often used in exercise rehabilitation programmes as they have been shown to be effective at reducing the pain associated with CLBP [25,27]. However CLBP patients must be careful not to perform exercises that result in pain, especially when stretching the flexors and extensors of the trunk and hips [50].

#### 4.3.1. Flexibility Programmes

A 4-week intervention programme involving 40 female NSCLBP patients between 45 and 65 years [27] included 10 exercises for the lumbo-pelvic spine to improve the lumbar flexibility and stability. The exercises were completed in positions which were non-weight bearing such as in a supine position, side lying and prone and were completed twice a week with 10 repetitions of each exercise.

The study reported a 54% significant increase for lumbar flexion and 98% for lumbar extension. Back pain also significantly improved by 58%. The results suggested that completing exercises to improve lumbar flexion and extension is important in reducing NSCLBP in women. This is because during the baseline measurements lumbar flexion was found to be correlated with back pain (*r* = −0.581). However these results cannot be generalised to men.

Lumbar extension exercises can reduce tension in the posterior annular fibers, and alter intradiscal pressure which allows anterior migration of the nucleus pulposus [115] which is important for the vertebral disc to withstand compression [118]. In addition lumbar flexion exercises stretch the hip flexors and lumbar extensors, and decrease the compressive forces on the posterior disc [115].

A further study researched the effect of a 6-week Pilates programme on hamstring and lower back flexibility and CLBP [25]. The study involved 34 NSCLBP patients aged 18–60 years, and were randomly assigned to either the Pilates group or the control group. The Pilates exercises were completed during a one hour class each week taught by a certified Pilates Institute Instructor, and two 30 min sessions each week at home without any supervision. The control group did not participate in the Pilates exercises and continued with their normal PA levels.

The study identified that flexibility significantly increased by 52.9% in the Pilates group, compared to a 7.8% increase in the control group which was not significantly different. Back pain also significantly decreased by 18.5% in the Pilates group, and there was no change in the back pain for the patients in the control group. The results suggested that Pilates exercises can significantly improve back pain and hamstring and lower back flexibility for NSCLBP patients.

The relationship between low back pain, lumbar flexion and hamstring flexibility was researched in a study involving 26 male University rowers who participated in rowing training six times a week [119]. Participants were assigned into groups according to whether they were currently suffering from low back pain (acute, sub-acute or chronic), had suffered from low back pain at some point in their lives or had never suffered from low back pain.

The study reported that the participants with current low back pain (11 participants) had a significantly reduced lumbar flexion compared to the participants without current low back pain (15 participants). However no significant difference was identified in hamstring flexibility between the two groups. In addition no significant difference was identified in lumbar flexion or hamstring flexibility between the participants who had experienced low back pain at some point in their lives (21 participants), or had not experienced low back pain (5 participants).

The results suggested that hamstring flexibility was not associated with low back pain occurrence, and therefore improving hamstring flexibility is not important for preventing low back pain or for an intervention programme for a patient with low back pain. Although, the results did highlight the importance of improving lumbar flexion for patients with current low back pain, and also suggested that a reduced lumbar flexion is an important factor for an occurrence of low back pain.

However the study [119] did not specifically focus on CLBP patients as the participants reported any experiences of low back pain, current or previous, and therefore could have included a range of acute, sub-acute or CLBP. In addition, the results from the study were in contrast to findings previously discussed [25], which suggested that improving hamstring flexibility is important for reducing NSCLBP.

A 3-month intervention programme for 86 NSCLBP patients [26] investigated the effects of progressive therapeutic exercise on spinal and muscle flexibility and back pain by dividing the patients into three groups: intensive training group, home exercise group and the control group. Follow up measurements at 6 and 12 months after baseline tests was also conducted. The intensive training group and home exercise group completed seven exercises for various parts of the body using either gym equipment, such as pulleys and bar bells (intensive training group) or without the use of extra equipment (home exercise group). The control group maintained their normal PA levels throughout the duration of the study and did not participate in an organised exercise programme. However no information was provided on which exercises were completed.

Back pain significantly decreased post intervention by 44% in the intensive training group, 32% in the home exercise group and 39% in the control group. The 6-month follow up identified that back pain had decreased further in the home exercise group to a 47% reduction which was significantly different to baseline. The intensive training group which increased compared to post intervention to a 32% reduction compared to baseline, although this was still significantly different. The control group also increased compared to post intervention to 28% and was not significantly different to baseline.

The flexibility of the hamstrings significantly increased at post intervention from 87°–90° in the intensive training group and 83°–87° in the home exercise group. However at the 12-month follow up hamstring flexibility had reduced to 83° in the intensive training group and 82° in the home exercise group. This suggests the importance of maintaining exercise which is aimed at improving flexibility, as both exercise groups lost the improved degree of hamstring flexibility. Although there was no correlation between back pain and flexibility which suggested the importance of other factors on back pain, such as an increase in core strength and aerobic fitness [16,19,23]. However the study [26] did not measure core strength or aerobic fitness. In addition the study [26] suggested improving flexibility could be important for preventing CLBP from occurring, as opposed to using exercises to improve flexibility as a rehabilitation from CLBP. See Table 3 for a summary of each of the discussed flexibility intervention programmes.

#### 4.3.2. Summary

Improving the flexibility of the lumbar spine and hamstrings can significantly reduce CLBP by 18.5%–58% [25,27]. This suggests the importance of including flexibility exercises in an intervention programme for CLBP patients. However no association between hamstring flexibility and low back pain was identified in male University rowers [119].

An improvement in lumbar flexibility can increase the range of motion of the spine, which can help to reduce back pain and assist with movement [112]. Hamstring muscle shortening reduces the hip flexion range of motion which impacts upon the lumbopelvic movement [114], and a decrease in the flexibility of the hip flexor and back extensor muscles can lead to lumbar lordosis, which can result in low back pain [87]. Therefore including lumbar flexion exercises in an intervention programme for CLBP is important, as lumbar flexion exercises stretch the hip flexors and lumbar extensors [115].

## 5. Conclusions

Exercise intervention programmes involving either muscular strength, flexibility or aerobic fitness is beneficial for NSCLBP but not acute low back pain. Non-specific acute low back pain patients recover in 4–6 weeks with or without a treatment [5], and exercising should be avoided to reduce the swelling of the affected area [39].

NSCLBP is multi factorial in nature [110,120], and no single exercise programme is optimal for all NSCLBP patients [50]. In addition, the most appropriate specific intervention for a NSCLBP patient is often unclear [12], and NSCLBP pain should not been considered as a homogenous condition meaning all cases are identical [121]. This suggests that a specific intervention programme focusing on one area of fitness for a group of NSCLBP patients may not be appropriate. This is a limitation of this review as the NSCLBP patients in the included studies may have responded differently to the exercise interventions. Consequently, a general exercise programme which combines muscular strength, flexibility and aerobic fitness would be beneficial for rehabilitation of NSCLBP. Further research is needed into the benefits of a combined exercise intervention programme involving muscular strength, flexibility and aerobic fitness for NSCLBP patients, as the literature has supported the use of each of these fitness areas individually, but more research should be conducted combining all three.

## Figures and Tables

**Table 1:** Aerobic exercise intervention programmes for NSCLBP patients.

Reference Number	Type of Population	Length of Intervention	Effect on Back Pain	Significance Levels
(Hoffman *et al.*, 2005) [63]	8 individuals with NSCLBP (4 male, 4 female)	25 min of cycle ergometry. 5 min at 50% peak oxygen uptake, then 20 min at 70% peak oxygen uptake	Pressure pain test. Pain significantly decreased by 28% at 2 min and 22% at 32 min post exercise compared to pre-exercise values. No gender/age differences in results	*p* < 0.05
(Shnayderman & Katz-Leurer, 2013) [16]	52 sedentary NSCLBP patients aged 18-65 years	Experimental group (walking on treadmill at 50% heart rate reserve). Control group: specific low back strengthening exercises. Both twice a week for 6 weeks	Low Back Pain Functional Scale: Significantly improved by 20% in experimental group and 15% in control group. No gender/age differences in results	*p* < 0.05
(Chan *et al.*, 2011) [15]	46 NSCLBP patients (10 male, 36 female)	8-week intervention. Both intervention and control groups received conventional physiotherapy. Intervention group only also prescribed aerobic exercise (40%–60% heart rate reserve)	Visual Analogue Scale (VAS): Intervention group: 47% significant reduction post intervention. Control: 42% significant reduction post intervention. No gender/age differences in results	*p* < 0.001
(Chatzitheodorou *et al.*, 2007) [17]	20 NSCLBP patients (11 male, 9 female). Excludes patients with BMI > 30	12-week intervention. Exercise group: high intensity aerobic exercise (running on treadmill at 85% of heart rate reserve). Control group: Passive treatment (ultrasound and did not include any form of PA)	McGill Pain Questionnaire. Exercise group: 41% significant reduction post intervention. Control: no significant change. No gender/age differences in results	*p* < 0.001
(Chatzitheodorou *et al.*, 2008) [18]	64 NSCLBP patients (26 male, 38 female) Excludes patients with BMI > 30	Patients randomly allocated into positive or negative dexamethasone suppression test. Both groups completed 12-week aerobic exercise programme (running on treadmill at 85% heart rate reserve)	McGill Pain Questionnaire. Positive suppression group: 30% significant reduction post intervention. Negative suppression group: 8% significant reduction post intervention. No gender/age differences in results	*p* < 0.001

**Table 2:** Muscular strength and stabilisation intervention programmes for NSCLBP patients.

Reference Number	Type of Population	Length of Intervention	Effect on Back Pain	Significance Levels
(Inani & Selkar, 2013) [19]	30 NSCLBP patients (20 male, 10 female) aged 20–50 years	3-month intervention. Experimental group: Completed core stabilization exercises including slow curl ups, bird dog, the plank and sit ups (raising head and shoulders off the ground with hands under the head). Control group: Completed conventional spine exercises including static stretching of muscles found to be tight	Visual Analogue Scale. Experimental group: 76.8% significant reduction post intervention. Control group: 62.8% significant reduction post intervention. No gender/age differences in results	*p* < 0.001
(Šarabon, 2011) [21]	10 NSCLBP patients (3 male, 7 female)	8-week core stability intervention programme involving activating core stability responses using unstable standing surfaces and unexpected movements of the upper limbs	Visual Analogue Scale. 39.5% significant reduction post intervention. No gender/age differences in results	*p* < 0.01
(Suni *et al.*, 2006) [23]	106 middle aged working men who had a reported episode of non-specific low back pain within the previous 3 months, but did not have severe disability	12-month programme in which participants exercised twice a week undergoing exercises to improve lumbar stability e.g., abdominal curl up with slight rotation and squat exercises. This exercise programme was combined with educating the patients on back pain and providing training on correct techniques for lifting.	Visual Analogue Scale. Significant 39% reduction	*p* < 0.01
(You *et al.*, 2014) [24]	40 NSCLBP patients (19 male, 21 female)	8-week stabilisation programme and follow up measurement after 2 months. Patients continued exercises throughout 2-month follow up period. Experimental group: Combined ankle dorsiflexion exercises (completed at 30% of maximal voluntary isometric contraction using resistance band for 10 sets of 20 s) with drawing in the abdominal wall. Control group: Drawing in the abdominal wall exercises alone	Experimental group, post intervention: Significant reduction of 32.5% (VAS), 23.2% (Pain Disability Index) and 21.5% (Pain Rating Scale). Control group, post intervention: Significant reduction of 16.8% (VAS), 12.4% (Pain Disability Index) and 8% (Pain Rating Scale). Experimental group, follow up measurement: Significant reduction of 46.8% (VAS), 39.2% (Pain Disability Index) and 30.7% (Pain Rating Scale) compared to pre intervention. Control Group, follow up measurement: Significant reduction of 38.7% (VAS), 18.8% (Pain Disability Index) and 14.6% (Pain Rating Scale) compared to pre intervention. No gender/age differences in results	*p* < 0.001
(Kim *et al.*, 2013) [20]	47 women with NSCLBP	Muscular strength 8-week intervention programme which investigated different angles of inversion traction on NSCLBP. Patients randomly allocated into 3 groups: supine, inversion −30° and inversion −60°. Each group completed a 3 min x 3 set inversion traction protocol at 0°, inverted −30° or inverted −60° for 4 days a week during 8 weeks	Visual Analogue Scale. Significant reduction of 61.6% in both inversion −30° and inversion −60° groups. Significant reduction of 34.9% in the supine group	*p* < 0.009
(Stankovic *et al.*, 2012) [22]	160 NSCLBP patients (63 male, 97 female) 18–75 years	4-week core muscular strength programme (control group) was compared to a core stability programme in addition to core muscular strength exercises (experimental group)	Experimental group: Significantly reduced by 35% post intervention. Control group: Significantly reduced by 14% post intervention. No gender/age differences in results	*p* < 0.001

**Table 3:** Flexibility intervention programmes for NSCLBP patients.

Reference Number	Type of Population	Length of Intervention	Effect on Back Pain	Significance Levels
(Masharawi & Nadaf, 2013) [27]	40 female NSCLBP patients between 45 and 65 years	Study group: Activities of daily living guidance and a 45 min group exercise session aimed at improving lumbar flexibility and stability. Exercise session was completed twice a week for 4 weeks with 10 repetitions of each exercise. Control group: Activities of daily living guidance only	Visual Analogue Scale. Study group: 58% significant improvement following intervention. Control group: no significant change	*p* < 0.001
(Gladwell *et al.*, 2006) [25]	34 NSCLBP patients aged 18–60 years	Pilates group: Completed Pilates exercises during a one hour class each week for 6 weeks, and two 30 min sessions each week at home without any supervision. Control group: Did not participate in the Pilates exercises and continued with their normal PA levels	Visual Analog Scale. Pilates group: 18.5% significant decrease following intervention. Control group: No significant difference. No gender/age differences in results	*p* < 0.05
(Kuukkanen & Malkia, 2006) [26]	86 NSCLBP patients	Intensive training group and home exercise group completed 3-month intervention programme: 7 exercises for various parts of the body using either gym equipment, such as pulleys and bar bells (intensive training group) or without the use of extra equipment (home exercise group). Control group: Maintained their normal PA levels and did not participate in an organised exercise programme	Intensive training: 44% significant reduction post intervention. Control: 39% significant reduction post intervention. Home exercise: 32% significant reduction post intervention. No gender/age differences in results	*p* < 0.05
